# Exosomes released from M2 macrophages transfer miR‐221‐3p contributed to EOC progression through targeting CDKN1B

**DOI:** 10.1002/cam4.3252

**Published:** 2020-06-26

**Authors:** Xiaoduan Li, Meiling Tang

**Affiliations:** ^1^ Department of Gynecology Shanghai First Maternity and Infant Hospital Tongji University School of Medicine Shanghai China

**Keywords:** CDKN1B, EOC, exosomes, M2 macrophages, miRNA

## Abstract

In contrast to other solid tumors within the abdominal cavity, epithelial ovarian cancers (EOCs) tend to undergo peritoneal metastasis. Thus, the peritoneal immune microenvironment is crucial for EOC progression. Previous reports indicate that the main immune cells within the peritoneum are M2 macrophages, specifically tumor‐associated macrophages (TAMs). The communication between TAMs and tumor cells plays an important role in EOC development, and exosomes, acting as micro–message carriers, occupy an essential position in this process. Microarray analyses of exosomes revealed that miR‐221‐3p was enriched in M2 exosomes. Furthermore, miR‐221‐3p suppressed cyclin‐dependent kinase inhibitor 1B (CDKN1B) directly. Thus, miR‐221‐3p contributed to the proliferation and G1/S transition of EOC cells. Additionally, low levels of CDKN1B were associated with EOC progression and poor prognosis. These observations suggest that TAMs‐derived exosomal miR‐221‐3p acts as a regulator of EOC progression by targeting CDKN1B. The results of this study confirm that certain exosomal microRNAs may provide novel diagnostic biomarkers and therapeutic targets for EOC.

## Graphical abstract

These observations propose TAMs‐derived exosomal miR‐221‐3p is one of the regulators in epithelial ovarian cancer (EOC) progression through targeting cyclin‐dependent kinase inhibitor 1B. These findings suggest that these exosomes or associated microRNAs might provide novel diagnostic biomarkers and treatments for EOC.

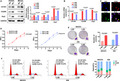

## 1 INTRODUCTION

Ovarian cancer is the most lethal gynecologic malignancy; however, the incidence of this cancer is quite low.^1^ Epithelial ovarian cancer (EOC) is the most common pathological type of ovarian cancer. In contrast to other tumors, EOC is prone to peritoneal metastasis, and tumor cells can be disseminated throughout the entire abdominal cavity by ascites.^2^ Refractory ascites can result in significant difficulties in EOC treatment, and thus, EOC is characterized by chemotherapy drug resistance and high recurrence.^3^ The peritoneum is the most common site for EOC colonization, and based on this, the immune microenvironment within the peritoneum is crucial for EOC progression. An immunosuppressive microenvironment further exacerbates tumor progression, and peritoneal EOC is characterized by immune deficiency. Previous studies have demonstrated that the main immune cells within the peritoneum are CD68^+^ macrophages, specifically tumor‐associated macrophages (TAMs).^4^

As a common component of the EOC tumor microenvironment, TAMs promote tumor development by secreting a number of growth factors and cytokines.^5^ Tumor‐associated macrophages are of the M2 phenotype that promote tumor progression and are associated with poor prognosis in numerous cancers.^6^ A number of research have revealed that TAMs are important for modifying the microenvironment and in promoting tumor growth by accelerating angiogenesis, modulating resistance, and suppressing the antitumor immune response.^7^

Exosomes are exocrine microvesicles possessing a diameter of 30‐150 nm, and they contain numerous types of proteins and nucleic acids.^8^ Cancer cells secrete a greater amount of exosomes than do normal cells, and more importantly, the inclusions of cancer exosomes are different.^9^ Thus, identifying the tumor‐specific exosomes or specific components of exosomes in body fluids can potentially contribute to the treatment and management of cancer. In support of this, some progress has been made in this research field, where it was observed that exosomes that were enriched in GPC1 and contained mutant KRAS mRNA could act as a reliable biomarker for pancreatic cancer detection.^10^ Exosomal microRNAs (miRNAs) are frequently regarded as potential diagnostic biomarkers of cancer.^11^ It has been reported that the level of miR‐19a in serum exosomes could act as a prognostic biomarker for recurrent colorectal cancer.^12^ Furthermore, certain specific exosomal miRNAs play an important role in modulating tumor growth, as evidenced by the ability of exosomes containing miR‐10b to promote the invasion of breast cancer cells.^13^ In this study, we examined miRNA microarrays of exosomes derived from M2, and we found that 42 miRNAs, including miR‐21‐5p, miR‐24‐3p, and miR‐221‐3p, were enriched in M2 exosomes.

CDKN1B is the gene that encodes the cell cycle regulator cyclin‐dependent kinase inhibitor 1B (CDKN1B) (p27). In general, CDKN1B is considered to act as a cell cycle progression inhibitor at G1/S transition. However, CDKN1B is not classified as a typical tumor suppressor protein, as very few mutations in CDKN1B have been reported in tumors.^14^ Nevertheless, based on advances in genomic analyses, CDKN1B has been found to be associated with tumor progression.^15^ Low expression of CDKN1B provides an advantageous prognostic marker in acute myeloid leukemia.^16^ Furthermore, a decline in CDKN1B levels is usually accompanied by miR‐221 or miR‐222 activities in certain tumors, including hepatocellular carcinomas and chronic lymphocytic leukemia.^17^, ^18^ Based on the previously mentioned microarray analysis and our results, miR‐221‐3p was selected as the targeted miRNA.

In this study, we revealed that TAM‐derived exosomes transferred miR‐221‐3p to EOC cells and induced the proliferation and migration of EOC cells through direct targeting of CDKN1B. Moreover, low levels of CDKN1B were associated with EOC poor prognosis. In this study, a new mechanism by which TAMs influence EOC was identified. Moreover, these exosomal miRNAs may provide novel therapeutic targets for EOC treatment.

## 2 MATERIALS AND METHODOLOGY

### 2.1 Clinical specimens

Specimens included 81 benign ovarian tumor patients and 114 EOC patients that were obtained from Shanghai First Maternity and Infant Hospital. None of the patients received chemotherapy. The purpose and possible risks of the study were explained to the patients, and written informed consent was obtained from each participant. The study protocol has been approved by the institutional review board of Shanghai First Maternity and Infant Hospital.

### 2.2 Cell culture

The SKOV3 and ID8 cell lines were purchased from Fuheng Bio (FH0135, FH1030, FuHeng Cell Center, Shanghai, China). DMEM (HyClone, SH30243.01B) supplemented with 10% fetal bovine serum (FBS, Gibco, 10099141) was used to culture the ID8 cells, and RPMI‐1640 (HyClone, SH30809.01B) supplemented with 10% FBS was used to SKOV3 cells. The cell lines were identified by short tandem repeats (STR) testing. The last STR identification was performed in August 2019.

### 2.3 M0 and M2 macrophages

Human peripheral blood mononuclear cells (PBMCs) were isolated from peripheral blood, and fresh human M0/M2 macrophages were isolated from PBMCs using CD14^+^ immunomagnetic beads (Miltenyi Biotec, 130‐050‐201) according to the manufacturer's instructions. Mouse M0/M2 macrophages were derived from C57 bone marrow. Fresh mouse peripheral blood monocytes were collected from C57 mice and isolated using F4/80 immunomagnetic beads (Miltenyi Biotec, 130‐110‐443) according to the manufacturer's instructions. M0 was induced by exposure to 10 ng/mL M‐CSF (Human & Mouse, RD System, 216‐MC‐010 & 416‐ML‐050). M2 was induced by treatment with 10 ng/mL M‐CSF and 20 ng/mL IL‐4 (Human & Mouse, RD System, 204‐IL‐020 & 404‐ML‐025).^19^

### 2.4 Exosome isolation

To isolate exosomes, M0/M2 macrophages were cultured in RPMI‐1640 supplemented with exosome‐depleted FBS (SBI, EXO‐FBS‐50A‐1) for 24‐48 hours. We then collected the supernatant and centrifuged twice (1000 *g* for 10 minutes and 3000 *g* for 30 minutes) to deplete the cell fragments. Next, Total Exosome Isolation Reagent was added to the cell culture medium (Life Technologies, 4478359) according to the manufacturer's instructions. The mixture was then centrifuged at 10 000 *g* for 1 hour after overnight treatment at 4˚C. The concentrations of the exosomes were detected using a bicinchoninic acid (BCA) Protein Assay Kit (Thermo, 23225).

### 2.5 Western blotting

The proteins for Western blotting (WB) were obtained by cell lysis using RIPA buffer followed by high‐speed centrifugation. Then the supernatant was collected to obtain the proteins, and all proteins were quantified using a BCA Kit (Thermo Scientific, 23225). The total protein obtained for each sample was 20 µg. The proteins were separated on SDS‐PAGE gels, and the proteins were then transferred to polyvinylidene fluoride membranes (Millipore, IPVH00010). These membranes were subsequently blocked using 5% nonfat milk for 1 hour. Finally, the membranes were incubated with glyceraldehyde‐3‐phosphate dehydrogenase (GAPDH) (CST, 5174), CD163 (Abcam, ab182422), CD206 (Abcam, ab125028), Arg1 (Abcam, ab124917), or CDKN1B (Abcam, ab32034) at 4°C overnight. Goat anti‐rabbit immunoglobulin G (IgG) (CST, 7074) was then used as the secondary antibody. The exosome biomarkers that were detected included CD63 (Abcam, ab216130), CD81 (Abcam, ab109201), and TSG101 (Abcam, ab125011). ImageJ was used to analyze the gray values of the blots, and GAPDH blots were used to calculate a ratio. The first experiment in the control group was then defined as the standard value used for quantitative analysis of the protein.

### 2.6 CCK8 and clone formation assay

SKOV3 (2 × 10^3^ cells/well) and ID8 (1.5 × 10^3^ cells/well) cells were seeded into 96‐well plates, and 10 μL of CCK8 plus 100 μL of solution reagent (Beyotime Biotechnology, C0041) was added to each well. After a 2 hours incubation at 37°C, the absorbance value was obtained at 450 nm using a 96‐well plate reader for Cell Counting Kit‐8 (CCK8).

For clone formation assays, SKOV3 (500 cells/well) and ID8 (500 cells/well) cells were seeded into six‐well plates and incubated at 37°C. The status of clones was observed every 3 days. After 12‐15 days of culture, the plates were fixed with 4% paraformaldehyde for 15 minutes and then stained with crystal violet for 15 minutes. The number of clones was determined using a microscope.

### 2.7 Flow cytometry for cell cycle detection

Epithelial ovarian cancers cells were collected into a centrifuge tube and washed with the precooled phosphate buffer saline (PBS). Then, 70% ethanol that was precooled in an ice bath was added and gently mixed at 4°C for 2 hours or more. Next, precooled PBS was used to wash the cells. Each tube containing cell samples were supplemented with 0.5 mL of propidium iodide staining solution (Beyotime, C1052), the cell precipitation was slowly and fully resuscitated, and the solutions were incubated at 37°C for 30 minutes in the dark. Flow cytometry was used to detect red fluorescence at the excitation wavelength of 488 nm.

### 2.8 Lentivirus transfection

Human/mouse miR‐221‐3p‐OE/KD lentiviruses were purchased from Shanghai Genechem Co, LTD, and specialized human/mouse miR‐221‐3p‐KD lentiviruses for use with suspension cells were used for macrophages. First, EOC cells were seeded onto the plate 1 day in advance to ensure that the cell density was 50%‐60% at the time of transfection. The virus was diluted to the desired concentration in fresh culture medium, and 5 μg/mL of polybrene was added to increase the infection efficiency. After 24 hours, the culture medium containing virus was removed, and new culture medium was added. The cells were cultured for 48‐72 hours and then screened using 2 μg/mL puromycin (Shanghai MaoKang Biotechnology, MS0011‐25MG).

For macrophage transfection, the cells were diluted to a 5 × 10^5^/mL suspension, and the virus was then added to the appropriate concentration. The supernatant containing the virus was removed by centrifugation after 12 hours, and it was then replaced with complete medium. The fluorescence expression of green fluorescent protein (GFP) was observed after culturing for 72 hours to confirm the infection efficiency.

### 2.9 Plasmid transfection

The CDKN1B plasmid was synthesized by Genomeditech (Shanghai) Co, LTD, and a negative control plasmid was also used for plasmid transfection. EOC cells were infected with lentivirus and screened as described above. The EOC cells were then passaged into a six‐well plate at a suitable density, and the cells were ready for transfection when they reached 70%‐80% confluency the next day. A total of 2 μg of plasmid was diluted into 50 μL of OPTI‐MEM (Gibco, 31985062) and mixed evenly, and 5 μL of Lipofectamine 3000 transfection reagent (Thermo, L3000001) was then added. Then the mixture was incubated at room temperature for 30 minutes. The prepared complex was then added to the cells along with OPTI‐MEM, and the cells were cultured for 6 hours. The transfection efficiency was then detected using fluorescence microscopy after a culture time of 18 hours.

### 2.10 Luciferase reporter assay

The 3ʹ‐UTR (untranslated regions) sequence of CDKN1B was amplified by PCR and then cloned into the experimental vector (GV272 vector, XbaI/XbaI enzyme digestion, purchased from Shanghai Genechem Co., LTD). CDKN1B wild‐type (WT) and mutated luciferase reporter plasmids were subsequently constructed. Additionally, hsa‐miR‐221‐3p or hsa‐miR‐NC were cotransfected with the GV272‐CDKN1B‐3ʹ‐UTR‐WT or the GV272‐CDKN1B‐3’‐UTR‐Mut into HEK‐293T cells, respectively. The luciferase activity in each group was measured using the Dual‐Luciferase Reporter Assay System (Promega, E1910) according to the manufacturer's protocol and then detected using a GloMax Detector.

### 2.11 Immunohistochemistry analysis

The tissue sections were incubated with CDKN1B antibody (Human & Mouse; Abcam, ab32034 & ab227911) overnight at 4°C. Next, HRP‐conjugated anti‐rabbit IgG (CST, 7074) was added and incubated with the sections for 60 minutes at 37°C. The sections were then washed three times for 5 minutes in PBS, and they were then incubated with 3,3’‐diaminobenzidine (DAB) for 30 seconds. Tissue microarrays were scanned using a Pannoramic MIDI (3D HISTECH) to acquire an immunohistochemistry (IHC) score that was used to evaluate the expression of CDKN1B.

### 2.12 Immunofluorescence detection

The primary antibodies used to detect CD163 and CD206 were CD163 (Abcam, ab156769) and CD206 (Abcam, ab125028), respectively. Cell nuclei were dyed using DAPI (Sigma, D9542). Alexa Fluor 488 conjugated anti‐mouse antibody (Jackson, 111‐545‐003) was used to detect CD163, and Cy3 conjugated anti‐rabbit antibody (Jackson, 705‐165‐003) was used to detect CD206. A Zeiss LSM510 laser confocal microscope was used to collect images.

### 2.13 In situ hybridization

The probe specific for hsa‐miR‐221‐3p was labeled with Cy3 and synthesized by Servicebio Biotechnology Co, LTD First, the sections were dewaxed using diethyl pyrocarbonate (DEPC) water, and they were then boiled in the repair solution for 10‐15 minutes and cooled naturally. Next, proteinase K (20 μg/mL) was added at 37°C for a digestion time of 10 minutes. The sections were then washed with PBS three times, and the prehybridization solution was subsequently added dropwise followed by incubation at 37°C for 1 hour. We then removed the prehybridization liquid and then added the hybridization liquid containing the hsa‐miR‐221‐3p probe to allow for hybridization overnight. Finally, DAPI dye solution was added to the sections, and they were incubated for 8 minutes in the dark. The excitation wavelength of Cy3 red light was 510‐560 nm, and the emission wavelength was 590 nm. The sections were observed using a Pannoramic MIDI (3D HISTECH) to acquire the mean fluorescence intensity (MFI) that was used to evaluate the expression of miR‐221‐3p.

### 2.14 In vivo experiment

ID8 is the EOC cell line derived from C57BL/6, and thus, these cells were suitable for use in C57BL/6 ovarian cancer model construction.^20^, ^21^ As described above, ID8‐NC/miR‐221‐3p‐OE/miR‐221‐3p‐KD cells were infected with lentivirus in a manner based on that used for the infection of ID8 cells. The WT ID8 cells were defined as ID8‐WT. The different groups were named according to the types of ID8 cells used or the treatment of mice. These groups included the ID8‐WT group, the ID8‐miR‐221‐3p‐OE group, the ID8‐WT group, the ID8‐WT + M2 exos group, and the ID8‐miR‐221‐3p‐KD group. C57BL/6 female mice (15‐17 g, 5‐6 weeks) were obtained from Shanghai Slac Laboratory Animal and raised in SPF conditions. A total of 30 mice were randomly divided into five groups.

The mice in the five groups were injected with different ID8‐luc cells (5 × 10^6^ cells/injection) into their orthotropic right ovaries using a micro‐syringe. At 14 days after the first injection, the ID8‐WT + M2 exos group was injected with 100 μL of PBS containing 50 μg of exosomes via intraperitoneal injection twice per week, while the ID8‐WT group was injected with an equivalent amount of PBS. The tumors in C57BL/6 mice were detected via luciferase expression using D‐luciferin (Invitrogen, 100 mg/kg), and images of mice were captured using a NightOWLⅡLB983 to assess tumor size weekly. IndiGo software was used to analyze the images. The experimental endpoint was 10 weeks after ID8 cell injection or upon death due to disease. Exosomes (0.5 μg/μL) were marked with 5 μmol/L of DIR Iodide (Yeasen, 40757ES25) and intraperitoneally injected into mice in the ID8‐WT group, and then IVIS Lumina LT Series III was used to record the biodistribution of exosomes in mice. All C57BL/6 mice were cared for according to the animal room management standards, and the in vivo experiments were approved by the Medical Animal Care of Tongji University.

### 2.15 Agilent miRNA microarray

The M2 macrophages were induced by exposure to 50 ng/mL of propylene glycol monomethyl ether acetate (PMA) (Sigma, P1585) and 20 ng/mL of IL‐4. Exosomes were collected as described above. RNAs derived from exosomes were extracted using a mir VanaTM RNA Isolation Kit (Applied Biosystem p/n AM1556). Exosomes derived from M0 and M2 macrophages were analyzed using an Agilent miRNA microarray.

### 2.16 Assessment of miRNA expression

The M0/M2 exosomes were collected and lysed using TRIzol (Invitrogen, 15596026). Then, RNAs were reverse‐transcribed into cDNAs (Qiagen, miScript II RT Kit, 218161). Then, the cDNAs were used for PCR detection (Qiagen, miScript SYBR Green PCR Kit, 218073). The miRNA primers for hsa‐miR‐221‐3p and mmu‐miR‐221‐3p were obtained from Shanghai GenePharma Co, Ltd. The fold change was calculated as 2‐ΔΔCt, where ΔΔCt = ΔCttreatment‐ΔCtcontrol and ΔCt = Cttarget gene‐CtU6.

### 2.17 Transfection of mimics and inhibitors

The mimics and inhibitors were obtained from Shanghai GenePharma Co, Ltd, The oligonucleotide sequences were 5ʹ (6‐carboxyfluorescein) FAM‐labeled, and fluorescence could be observed after successful transfection. We transferred the mimics or inhibitors into cells using X‐tremeGENE siRNA transfection reagent (Roche, 04476093001) according to the manufacturer's instructions. The efficiency of transfection was examined using RT‐PCR after 48 hours or by WB after 72 hours.

### 2.18 Assessment of mRNA expression

The samples were lysed using TRIzol, and RNAs were reverse‐transcribed into cDNA for PCR detection using an reverse transcription‐polymerase chain reaction (RT‐PCR) kit (Takara, DRRo14A). The RT‐PCR primers used for human CDKN1B in were (F) 5ʹ‐AACGTGCGAGTGTCTAACGG‐3ʹ and (R) 5ʹ‐CCCTCTAGGGGTTTGTGATTCT‐3ʹ. Those used for mouse CDKN1B were (F) 5ʹ‐TCAAACGTGAGAGTGTCTAACG‐3ʹ and (R) 5ʹ‐CCGGGCCGAAGAGATTTCTG‐3ʹ. Those used for human CDK2 were (F) 5ʹ‐CCAGGAGTTACTTCTATGCCTGA‐3ʹ and (R) 5ʹ‐TTCATCCAGGGGAGGTACAAC‐3ʹ. Those used for mouse CDK2 were (F) 5ʹ‐ATGGAGAACTTCCAAAAGGTGG‐3ʹ and (R) 5ʹ‐CAGTCTCAGTGTCGAGCCG‐3ʹ. Those used for human CDK4 were (F) 5ʹ‐ATGGCTACCTCTCGATATGAGC‐3ʹ and (R) 5ʹ‐CATTGGGGACTCTCACACTCT‐3ʹ. Those used for mouse CDK4 were (F) 5ʹ‐ATGGCTGCCACTCGATATGAA‐3ʹ and (R) 5ʹ‐TGCTCCTCCATTAGGAACTCTC‐3ʹ.

### 2.19 Statistics

SPSS21.0 software (SPSS Inc) was used for all statistical analyses. All figures are presented as the mean ± SEM. In vitro experiments were repeated three times at minimum. The univariate ANOVA (least significant difference [LSD] test), the Nonparametric test, and the Mann‐Whitney test were two‐tailed to calculate *P* values. Overall survival (OS) was calculated using the Kaplan‐Meier method. *P* < .05 was considered statistically significant.

### 2.20 Study approval

The human and animal study protocols were ratified by the Institutional Review Board of Shanghai First Maternity and Infant Hospital. All samples possessed written informed consent that was signed by participants or their guardians.

## 3 RESULTS

### 3.1 The supernatant from M2 macrophages contributed to the proliferation and G1/S transition of EOC cells

**FIGURE 1:**
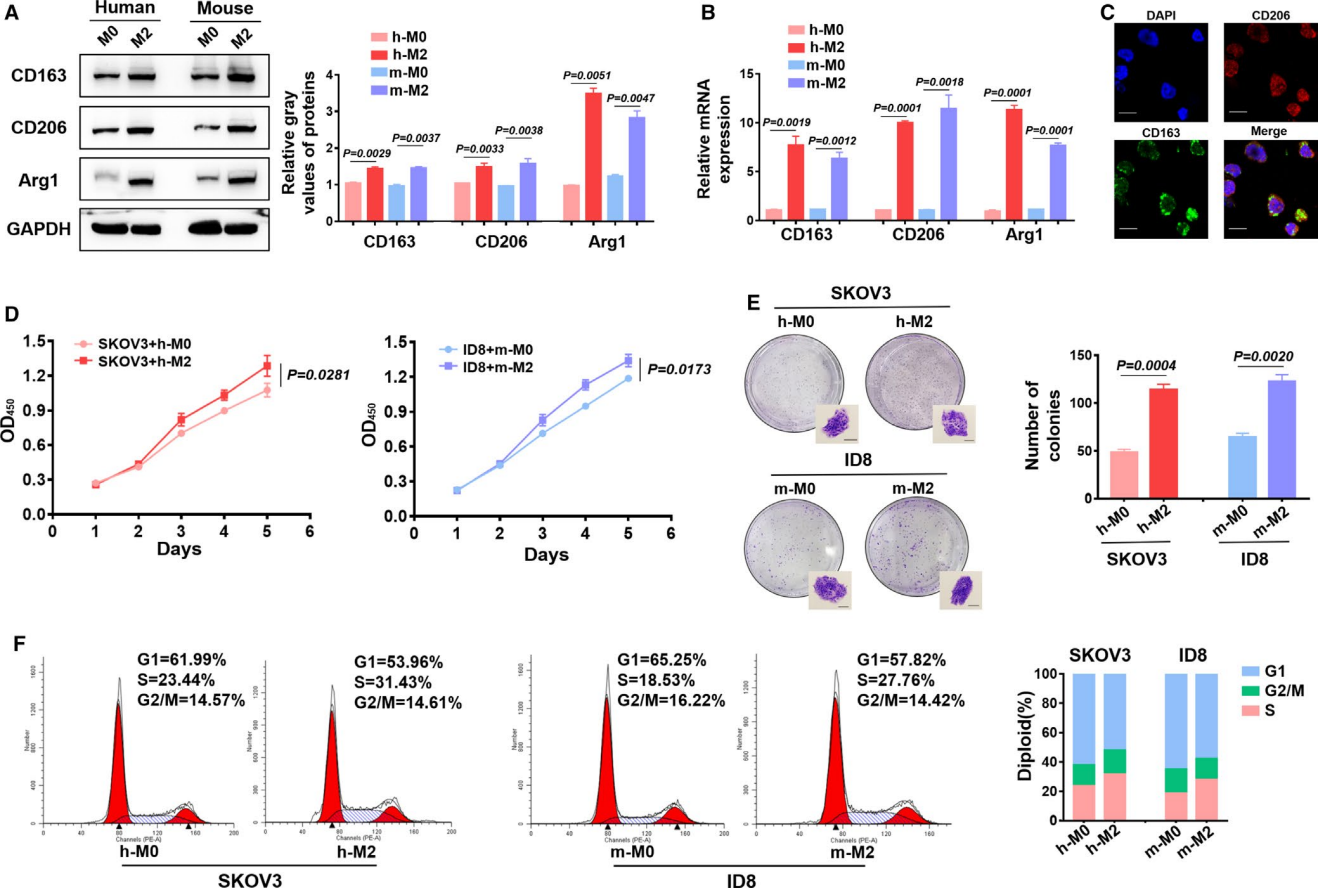
Effect of the supernatant from M2 macrophages on epithelial ovarian cancer (EOC) cells. A and B, Western blotting and RT‐PCR analyses were used to examine the M2‐phenotype markers CD163, CD206, and Arg1. C, Immunofluorescence staining revealed the membrane markers of M2 macrophages. Scale bar, 20 μm. D, CCK8 assays were used to examine the proliferation of EOC cells that were cocultured with the supernatant from M0/M2 macrophages. E, Clone formation experiments showed the amplification of EOC cells that were cocultured with the supernatant from M0/M2 macrophages. Scale bar, 200 μm. F, Flow cytometry confirmed the cell cycle changes in EOC cells that were cocultured with the supernatant from M0/M2 macrophages

Peripheral blood mononuclear cells were used as a source of human M0/M2 macrophages, and mouse M0/M2 macrophages were derived from C57 bone marrow. M‐CSF was used to induce M0 macrophages, while M‐CSF and IL‐4 were used to induce M2 macrophages. The TAM markers CD163, CD206, and Arg1 were analyzed by WB and RT‐PCR. Compared to expression levels in M0 macrophages, M2 macrophages possessed elevated expression of CD163, CD206, and Arg1 (Figure 1A,B). Immunofluorescence staining revealed that M2 macrophages possessed membrane expression of CD163 and CD206 (Figure 1C). To further investigate the characteristic function of EOC cells after exposure to M2 macrophages, the supernatant from M0/M2 macrophages were collected and added to EOC cells. CCK8 analyses revealed that SKOV3 cells proliferated at a faster rate after coculture with M2 supernatant than those that were cocultured with M0 supernatant. Additionally, the ID8 cells that cocultured with mouse M2/M0 supernatant also exhibited the same phenomenon (Figure 1D). The clone formation experiments also revealed that EOC cells cocultured with M2 supernatant formed a greater number of colonies (Figure 1E). Flow cytometry analyses confirmed that the supernatant from M2 macrophages promoted G1/S transition in both human and mouse cell lines (Figure 1F).

### 3.2 Exosomes derived from M2 macrophages were a crucial component of the supernatant and contributed to EOC cell proliferation and G1/S transition

**FIGURE 2:**
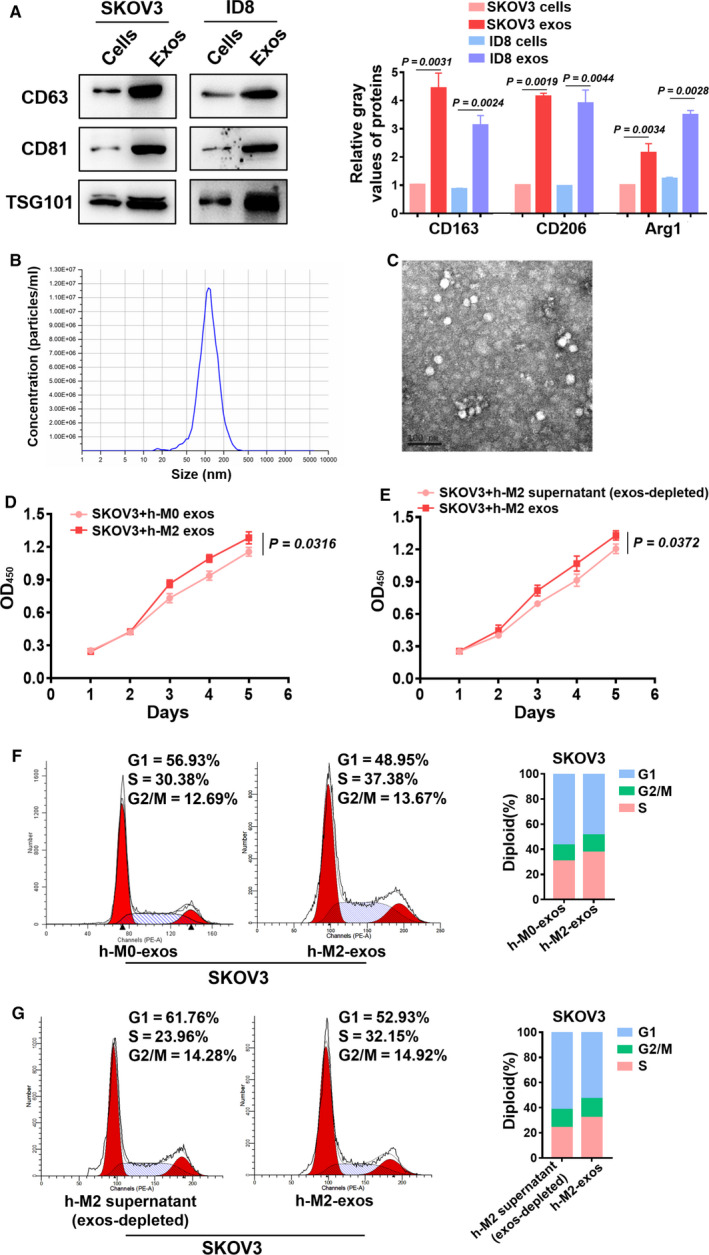
Exosomes present in the supernatant played important roles in promoting SKOV3 cell proliferation and G1/S transition. A, Exosome markers CD63, CD81, and TSG101 were identified by Western blotting. B, NTA analyses showed the size of exosomes. C, The typical morphology was identified by transmission electron microscopy. Scale bar, 100 nm. D, CCK8 showed the proliferation of SKOV3 cells that were cocultured with M2/M0 exosomes. E, CCK8 assays were used to examine the proliferation of SKOV3 cells that were cocultured with M2 exosomes or exosome‐depleted supernatant. F, Flow cytometry confirmed the G1/S transition of SKOV3 cells that were cocultured with the exosomes derived from M0/M2 macrophages. G, Flow cytometry confirmed the G1/S transition of SKOV3 cells that cocultured with M2 exosomes or exosome‐depleted supernatant

The proteins from exosomes derived from M2 macrophages were obtained following exosome lysis and identified by WB using antibodies specific for the exosomal markers CD63, CD81, and TSG101 (Figure 2A). The diameter of exosomes ranged from 30 to 150 nm according to nanoparticle tracking analysis (NTA) (Figure 2B), and the typical morphology was identified by transmission electron microscopy (Figure 2C). To further confirm that the exosomes and not the cytokines derived from M2 macrophages were the active component, the exosomes derived from M0/M2 macrophages were collected and then depleted from the supernatant by ultracentrifugation. CCK8 analyses indicated that SKOV3 cells proliferated faster after the additional of M2 exosomes than they did after the addition of M0 exosomes (Figure 2D). SKOV3 cells treated with M2 exosomes also proliferated faster than SKOV3 cells treated with exosome‐depleted supernatant (Figure 2E). Additionally, the ID8 cells exhibited a similar phenomenon (Figure S1A,B). Flow cytometry analysis also confirmed that the exosomes from M2 macrophages promoted G1/S transition in both human and mouse EOC cells (Figure 2F; Figure S1C), and M2 exosomes promoted the G1/S transition of EOC cells to a greater extent than did treatment with exosome‐depleted supernatant (Figure 2G; Figure S1D).

### 3.3 MiR‐221‐3p was enriched in the M2 exosomes and played an important part in promoting EOC cell proliferation and G1/S transition

**FIGURE 3:**
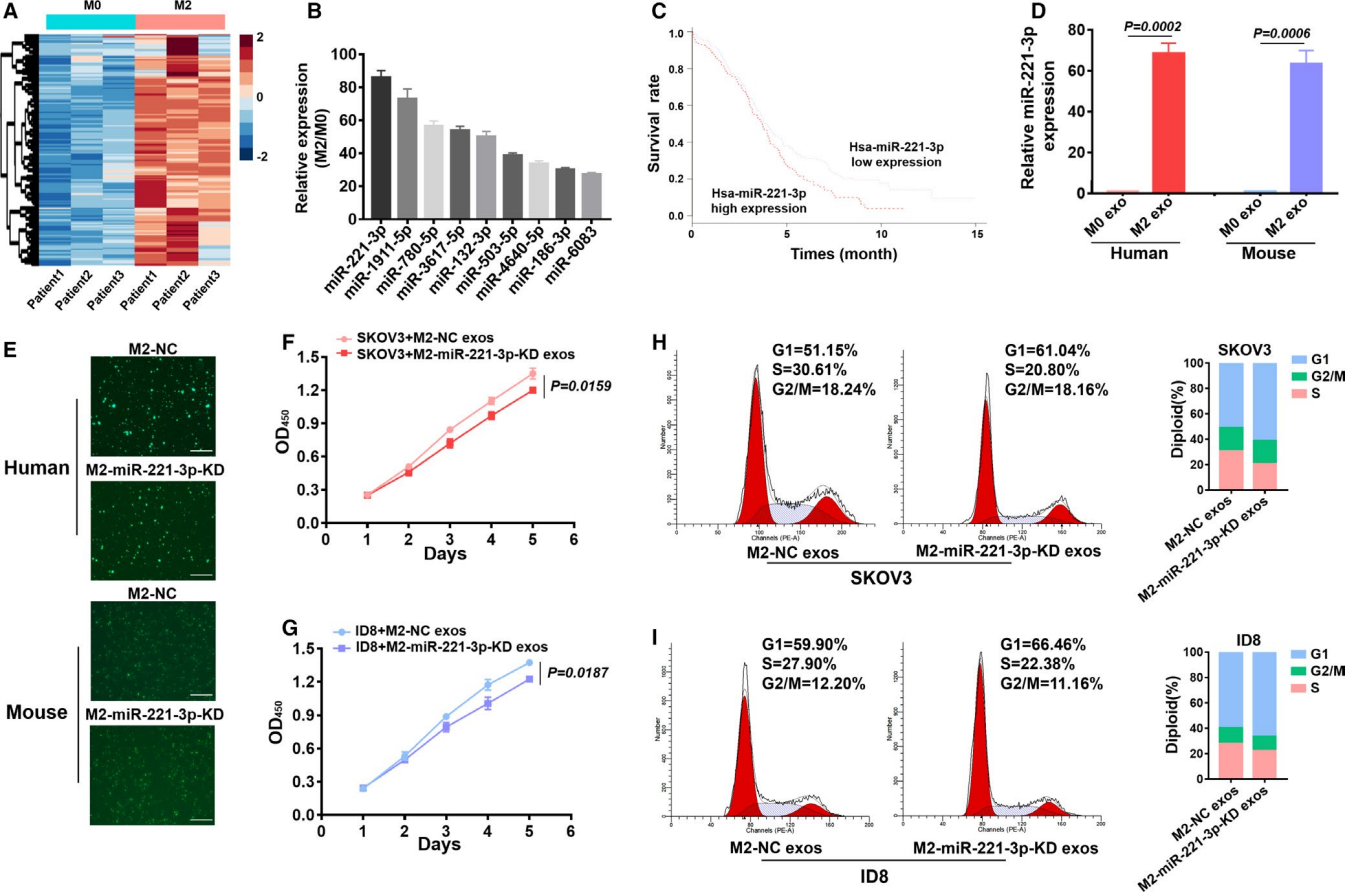
MiR‐221‐3p was enriched in the M2 exosomes and played an essential role in epithelial ovarian cancer (EOC) progression. A, Agilent microRNA (miRNA) microarrays were used to profile miRNAs in exosomes released by M0 and M2 macrophages. B, The top nine miRNAs that were related to cell cycle were shown. C, The relationship between miR‐221‐3p expression and OS of EOC patients according to the TCGA database. D, RT‐PCR confirmed the high expression of hsa‐miR‐221‐3p in human M2 exosomes and mmu‐miR‐221‐3p in mouse M2 exosomes. E, Fluorescence microscopy revealed the transfection efficiency of miR‐NC/miR‐221‐3p‐KD lentivirus in M2 cells. Scale bar, 200 μm. F and G, CCK8 assays were used to assess the proliferation and G1/S transition of EOC cells that were cocultured with M2‐NC/M2‐miR‐221‐3p‐KD exosomes. H and I, Flow cytometry showed the G1/S transition of EOC cells that were cocultured with M2‐NC/M2‐miR‐221‐3p‐KD exosomes

As mentioned above, exosomal miRNAs are important for intercellular communication. Therefore, we used miRNA microarrays to analyze different miRNAs in exosomes derived from M2 macrophages. The results revealed that 42 miRNAs were upregulated in M2 macrophage‐secreted exosomes (Figure 3A). A number of these miRNAs were related to cell cycle function, and the top nine miRNAs are shown (Figure 3B). Among these, hsa‐miR‐221‐3p was upregulated 87.79 ± 9.0‐fold (*P* = .0011, nonparametric *t* test). Furthermore, we analyzed the miR‐221‐3p expression of EOC patients in the TCGA database. High miR‐221‐3p expression levels were associated with poor OS according to data from the TCGA database (*P* = .0090, Mantel‐Haenszel test; Figure 3C). We collected exosomes from macrophages, and we found that hsa‐miR‐221‐3p was enriched in human M2 exosomes, and similarly, mmu‐miR‐221‐3p was enriched in mouse M2 exosomes (Figure 3D).

To identify the crucial role of miR‐221‐3p in macrophage exosomes, we transfected macrophages with lentivirus to knock down the expression of miR‐221‐3p. The observed GFP fluorescence confirmed the transfection efficiency (Figure 3E). Compared to the NC group, exosomes that contained miR‐221‐3p‐KD could inhibit EOC cell proliferation (Figure 3F,G). Flow cytometry results also indicated that the miR‐221‐3p‐KD group inhibited EOC cell G1/S transition (Figure 3H,I).

### 3.4 Alterations in miR‐221‐3p in EOC cells influenced tumor cell proliferation and G1/S transition

**FIGURE 4:**
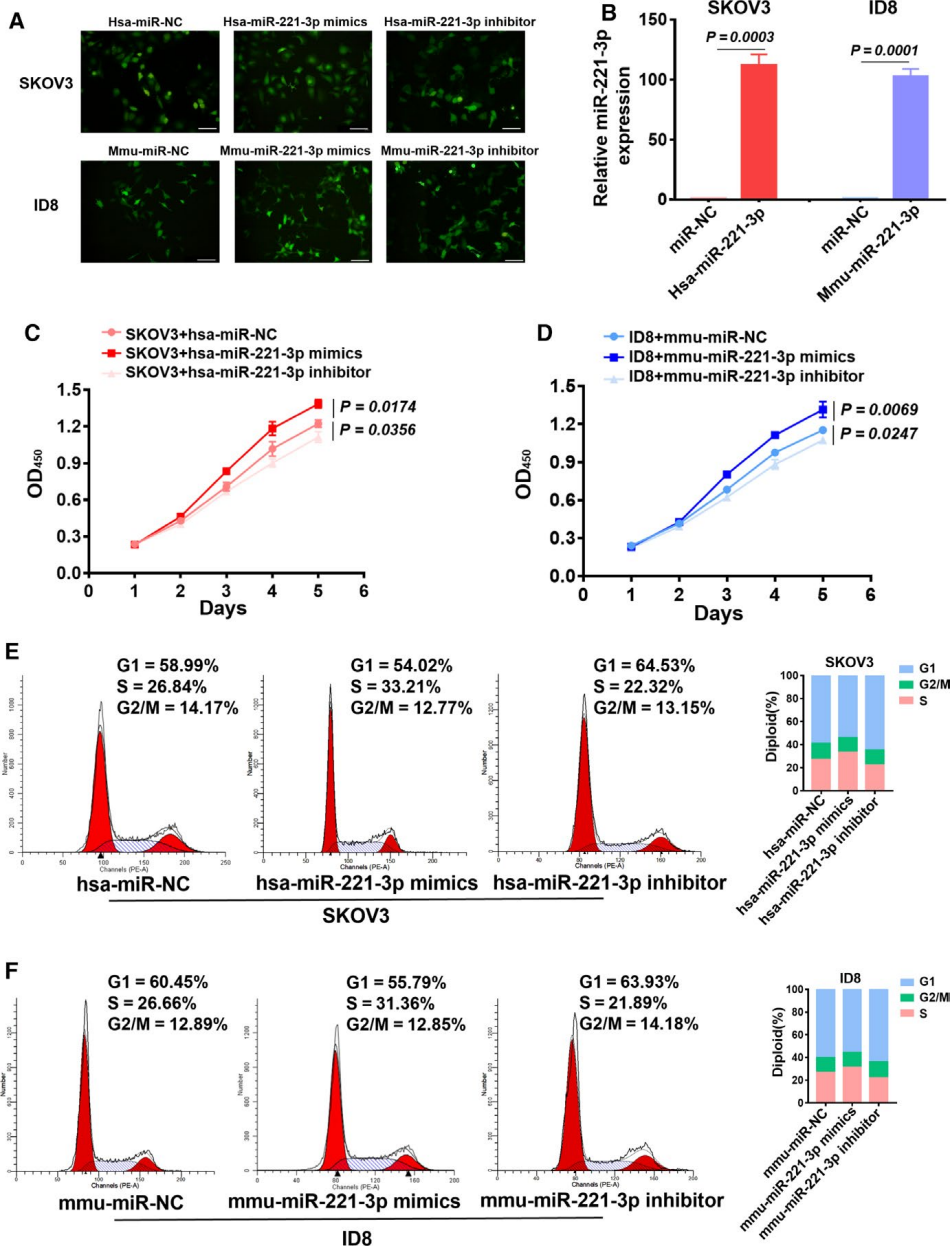
Changes in miR‐221‐3p in tumor cells directly influenced epithelial ovarian cancer (EOC) progression. A, Fluorescence microscopy showed the transfection efficiency of miR‐NC/miR‐221‐3p mimics/miR‐221‐3p inhibitors. Scale bar, 200 μm. B, RT‐PCR confirmed the transfection efficiency of miR‐NC/miR‐221‐3p mimics. C, CCK8 assays were used to assess the proliferation of SKOV3 cells that were treated with hsa‐miR‐NC/hsa‐miR‐221‐3p mimics/hsa‐miR‐221‐3p inhibitors. D, CCK8 assays were performed to assess the proliferation of ID8 cells that were treated with mmu‐miR‐NC/mmu‐miR‐221‐3p mimics/mmu‐miR‐221‐3p inhibitors. E and F, Flow cytometry was used to assess the G1/S cell cycle transition of EOC cells that were transfected with the miR‐NC/miR‐221‐3p mimics/miR‐221‐3p inhibitors

To explore the effect of miR‐221‐3p on EOC cells, we expressed the hsa‐miR‐NC/hsa‐miR‐221‐3p mimics/hsa‐miR‐221‐3p inhibitor in SKOV3 cells and the mmu‐miR‐NC/mmu‐miR‐221‐3p mimics/mmumiR‐221‐3p inhibitor in ID8 cells. Fluorescence microscopy analysis revealed that there was high transfection efficiency at 18 hours (Figure 4A). RT‐PCR was used to confirm the transfection efficiency of the miR‐221‐3p mimics (Figure 4B). CCK8 assays were performed daily to assess the proliferation of EOC cells for 5 days. As shown in the results, SKOV3 cells treated with hsa‐miR‐221‐3p mimics exhibited faster proliferation compared to that in cells treated with hsa‐miR‐NC. The hsa‐miR‐NC group also proliferated more rapidly than did the hsa‐miR‐221‐3p inhibitor group (Figure 4C). Similarly, mmu‐miR‐221‐3p mimics promoted proliferation of ID8 cells, and the mmu‐miR‐221‐3p inhibitor suppressed proliferation in these cells (Figure 4D). Additionally, flow cytometry analyses indicated that transfection of EOC cells with the miR‐221‐3p mimics promoted G1/S transition in both human and mouse cell lines, and the opposite result was observed in the miR‐221‐3p inhibitor group (Figure 4E,F).

### 3.5 MiR‐221‐3p contributed to EOC cell proliferation and G1/S transition through targeting CDKN1B

**FIGURE 5:**
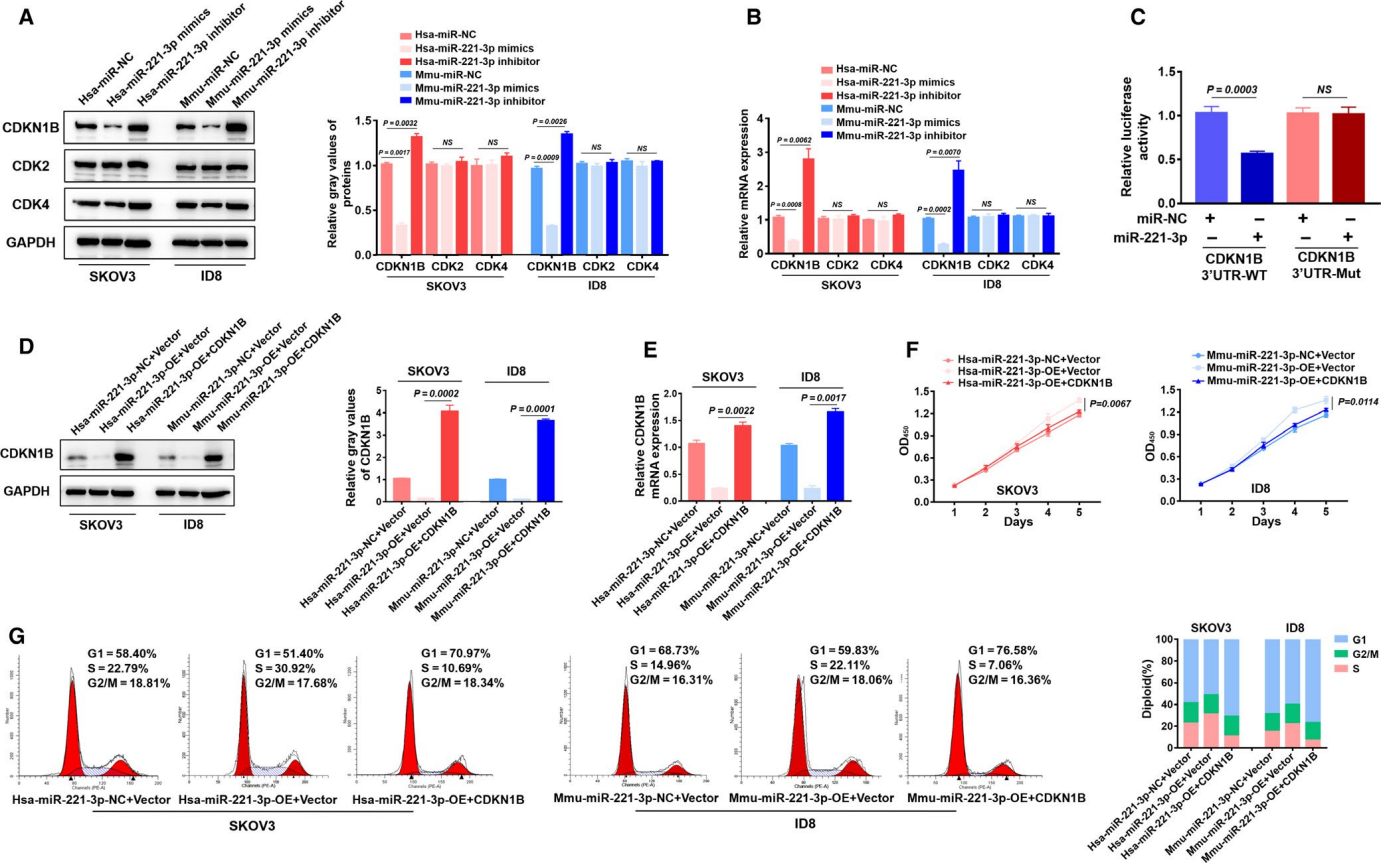
MiR‐221‐3p affected epithelial ovarian cancer (EOC) cells by targeting CDKN1B. A and B, Western blotting and RT‐PCR analyses were used to examine CDKN1B, CDK2, and CDK4 expression after transfecting EOC cells with miR‐NC/miR‐221‐3p mimics/miR‐221‐3p inhibitors. C, The luciferase reporter assay confirmed that miR‐221‐3p could bind to the CDKN1B 3ʹUTR. D and E, On the basis of miR‐221‐3p‐OE, supplementation with CDKN1B offset the CDKN1B expression level in SKOV3 and ID8 cells. F, Supplementation with CDKN1B offset the proliferation effect of the overexpression of miR‐221‐3p mimics in SKOV3 and ID8 cells. G, Supplementation with CDKN1B offset the G1/S transition of the overexpression of miR‐221‐3p mimics in SKOV3 and ID8 cells

To further elucidate the cell cycle protein that was affected by miR‐221‐3p, we transfected hsa‐miR‐NC, hsa‐miR‐221‐3p mimics, and hsa‐miR‐221‐3p inhibitors into SKOV3 cells and mmu‐miR‐NC/mmu‐miR‐221‐3p mimics/mmu‐miR‐221‐3p inhibitors into ID8 cells. Then, we detected if the cell cycle protein expression was altered after transfection. Western blotting and RT‐PCR analyses revealed that CDKN1B expression was downregulated after transfection with the miR‐221‐3p mimics, while the expression level of this protein was upregulated after transfection with the inhibitor. Despite this, there was no significant change in CDK2 and CDK4 expression levels (Figure 5A,B). Analysis of the potential targets of miR‐221‐3p revealed that it can bind to the CDKN1B 3ʹUTR, and the luciferase analysis report confirmed these results (Figure 5C).

To explore if CDKN1B is the key downstream factor influenced by miR‐221‐3p, EOC cells were infected with miR‐NC/miR‐221‐3p‐OE lentivirus, and on the basis of miR‐NC/miR‐221‐3p‐OE, we transfected CDKN1B or NC plasmid in the SKOV3 and ID8 cells. Supplementation with CDKN1B offset the effects of miR‐221‐3p overexpression in SKOV3 and ID8 cells. These effects included alterations in CDKN1B expression level (Figure 5D‐5E), proliferation (Figure 5F), and G1/S transition (Figure 5G).

### 3.6 MiR‐221‐3p contributed to EOC progression in vivo, and CDKN1B affected prognosis in EOC patients

**FIGURE 6:**
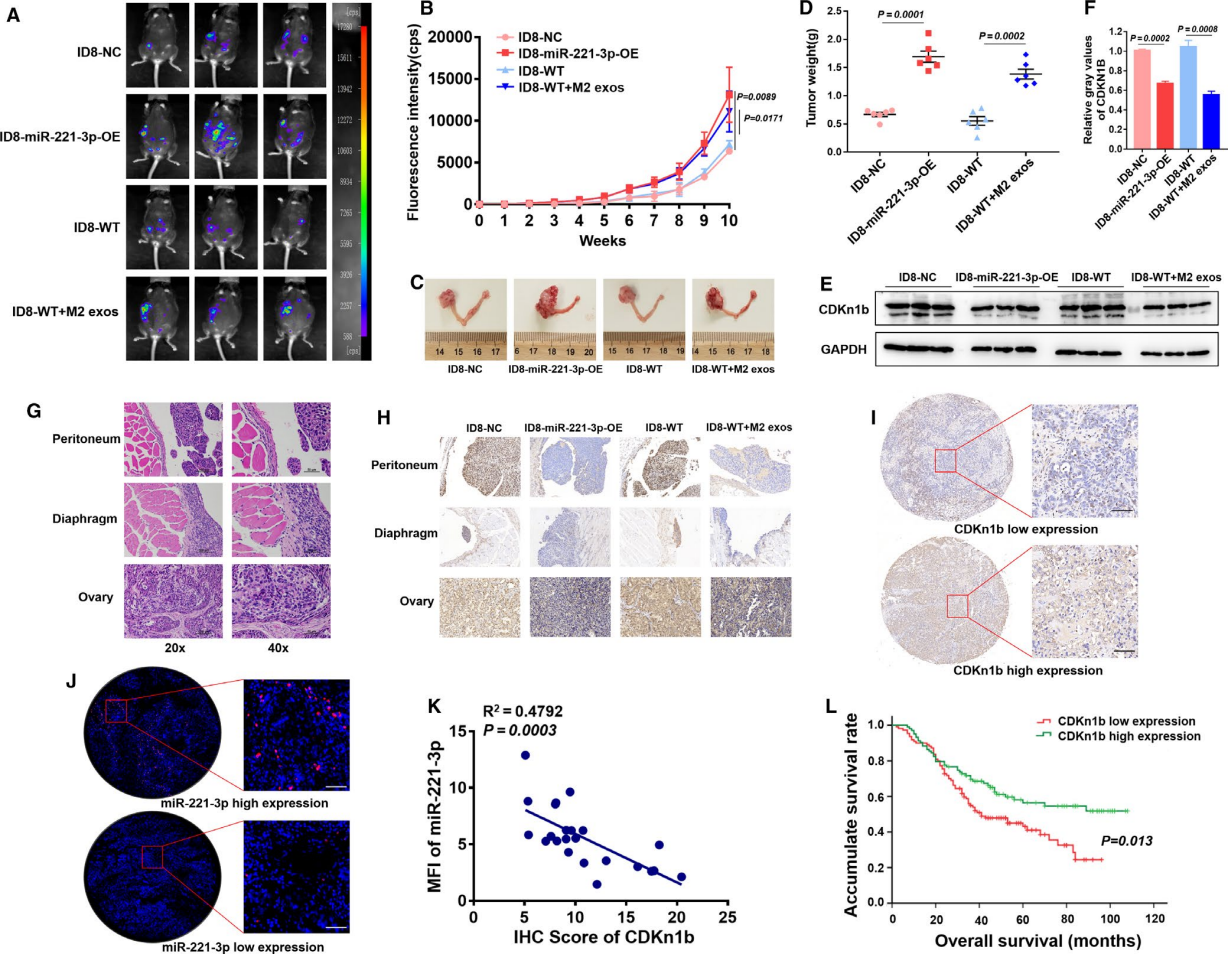
M2 exosomes and miR‐221‐3p contributed to epithelial ovarian cancer (EOC) progression in vivo, and CDKN1B affected prognosis in EOC patients. A, A bioluminescence imaging system was used to assess tumor growth, and the typical images at week 10 are presented. B, There was an increasing trend in tumor fluorescence intensity (cps) in the four groups after injection of ID8 cells at 1‐10 wk. C, Typical images of ovarian tumors after mice were sacrificed. D, The tumor weights for the ID8‐NC, ID8‐miR‐221‐3p‐OE, ID8‐wild‐type (WT), and ID8‐WT + M2 exos groups after the mice were sacrificed are shown. E and F The tumor tissues from different groups were collected for Western blotting analysis to detect CDKN1B expression. G, Hematoxylin‐eosin staining of the peritoneum, diaphragm, and ovarian from sacrificed mice in the ID8‐WT group. 40×, Scale bar, 50 μm. H, The tissues from different groups were collected for immunohistochemistry (IHC) Immunohistochemistry (IHC) to detect CDKN1B distribution and expression. I, IHC was used to evaluate the expression of CDKN1B in tissue microarray. Scale bar, 50 μm. J, In situ hybridization was used to detect the expression level of miR‐221‐3p in tissue. Scale bar, 50 μm. K, The MFI of miR‐221‐3p and the IHC score of CDKN1B was inversely correlated. L, The relationship between low CDKN1B expression and poor prognosis among 81 benign ovarian tumor patients and 114 EOC patients

To investigate the role of miR‐221‐3p in EOC progression in vivo, mice were divided into different groups according to treatment, and these groups included the ID8‐NC group, the ID8‐miR‐221‐3p‐OE group, the ID8‐WT group, the ID8‐WT + M2 exos group, and the ID8‐miR‐221‐3p‐KD group After injecting the right ovary of each mouse orthotopically with different ID8‐luc cells, the ID8‐WT + M2 exos group was injected M2 exosomes intraperitoneally twice every week. The in vivo biodistribution of the intraperitoneally injected exosomes is shown in Figure S2A. A bioluminescence imaging system was used to assess tumor progression and to allow the flux (cps) of tumors in different mice to be recorded each week. Images of typical mice images at week 10 are shown (Figure 6A). The ID8‐miR‐221‐3p‐OE group (15.023 ± 1.412 × 10^3^ cps) exhibited a more enhanced tumor flux compared to that of the ID8‐NC group (6.917 ± 0.263 × 10^3^ cps), and the ID8‐WT + M2 exos group (14.852 ± 0.737 × 10^3^ cps) possessed a more enhanced tumor flux compared to that of the ID8‐WT group (6.832 ± 0.112 × 10^3^ cps) (Figure 6B). Compared to the ID8‐NC group, the ID8‐miR‐221‐3p‐KD group exhibited a decreasing trend; however, no significant difference was observed (Figure S2B). After the mice were euthanized, tumor tissues were removed and weighed. The tumor weight was consistent with the tumor flux results (Figure 6C,D; Figure S2C). The tumor tissues were then collected for WB analysis, and the results indicated that the expression levels of CDKN1B in the ID8‐miR‐221‐3p‐OE group and the ID8‐WT + M2 exos group were significantly lower (Figure 6E,F). We also observed that the metastases were focused within the peritoneum and diaphragm, and hematoxylin‐eosin staining confirmed the tumor characteristics (Figure 6G; Figure S2D). Immunohistochemistry sections were also used to assess the CDKN1B expression levels in the ovaries, peritoneums, and diaphragms in different groups, and these expression levels exhibited a similar trend to those observed in the WB analysis (Figure 6H). Together, these data indicate that miR‐221‐3p overexpression and the M2 exosomes promoted EOC progression in vivo.

Previous studies have found that CDKN1B was related to EOC progression, and thus, we analyzed the expression of CDKN1B in clinical specimens. Tissue microarray analyses included 81 benign ovarian tumor tissues and 114 EOC tissues as estimated by IHC (Figure 6I). Also, in situ hybridization incorporating miR‐221‐3p probes was used to detect the expression levels of miR‐221‐3p in tissues (Figure 6J). We used IHC scores to detect the expression of CDKN1B, and we evaluated the expression of miR‐221‐3p using the MFI. The MFI of miR‐221‐3p was inversely related to the IHC score of CDKN1B in the tissue microarray (Figure 6K). Patients with low CDKN1B expression exhibited a worse prognosis than those with high expression (Figure 6L). Therefore, the results suggested that low expression of CDKN1B may be related to EOC progression. The clinical and pathological characteristics of all patients are presented in Table 1.

**TABLE 1:** Univariate and multivariable analyses of OS in 114 EOC patients

Variables	N	Overall survival (OS) univariate analysis (mean ± SEM)	*P*‐value
Age			
<50	46	50.25 ± 3.12	.107
≥50	68	48.72 ± 4.43	
Clinical stage			
I + II	29	60.44 ± 4.13	.002
III + IV	85	36.51 ± 2.92	
Histological subtype			
Serous ovarian carcinoma (SOC)	81	40.34 ± 2.57	.029
Non serous ovarian carcinoma (NSOC)	33	51.27 ± 4.36	
Histological grade			
Low	30	61.52 ± 3.72	.011
High	84	35.21 ± 2.93	

Abbreviation: EOC, epithelial ovarian cancer.

## 4 DISCUSSION

Exosomes play a crucial part in the communication between TAMs and tumor cells, and they contain numerous miRNAs, mRNAs and proteins.^11^, ^22^ Exosomes contents function as signaling molecules between cancer cells and the microenvironment. Both tumor cell exosomes and stromal cell exosomes can affect all stages of cancer progression.^23^ For example, noncoding RNAs derived from exosomes can be transmitted to monocytes, altering them into cancer‐related monocytes that can contribute to immune escape via PD‐L1 expression.^24^ The exosomes derived from tumors play multifaceted roles in promoting metastasis by regulating proliferation, invasion, and angiogenesis.^25^ Here, we found that the exosomes derived from M2 macrophages contributed to EOC cell proliferation and G1/S transition. In vivo experiments also indicated that exosomes derived from M2 cells contributed to EOC progression.

Exosomes function through the activity of their contents, and of these, miRNA is one of the most important. Transfer of exosomal miR‐25‐5p to hepatocellular carcinoma cells (HCC) significantly enhanced their capacity for migration and invasion, while the inhibition of miR‐25‐5p can mitigate these effects on circulating tumor cells.^26^ Additionally, myeloma cells regulate the expression of miR‐27b‐3p and miR‐214‐3p in fibroblasts through the release of exosomes, and the elevation of miR‐27b‐3p and miR‐214‐3p can promote the expression of fibroblast activation markers.^27^ In this study, we used miRNA microarrays to identify distinct miRNAs in exosomes derived from M2 macrophages. We found that miR‐221‐3p is the most specifically expressed of miRNAs related to cell cycle. Furthermore, high miR‐221‐3p expression levels were related to poor OS in EOC patients according to the TCGA database. MiR‐221‐3p contributed to EOC cell proliferation and G1/S transition through targeting of CDKN1B. Knockdown of miR‐221‐3p alone in ID8 cells exerted no significant effect compared to that observed with the ID8‐NC group in vivo, indicating that the mechanism of miRNA‐mediated inhibition is relatively complex in vivo. Further research should be conducted to examine the blocking mechanism of miRNAs if they are to be used for clinical treatment.

CDKN1B (P27) is a cell cycle–dependent kinase inhibitor that has been studied extensively. It influences the regulation of the cell cycle in a number of ways, inhibits cell division and proliferation, and also promotes cell differentiation and apoptosis. CDKN1B plays a vital role in the development, drug resistance, treatment, and prognosis of cancer.^16^, ^28^ CDKN1B is downregulated in different tumors according to a number of studies, and based on this, this protein has been identified as a noncanonical tumor suppressor gene.^29^ In advanced head and neck cancer, exosomes from cancer‐associated fibroblasts promoted tumor cisplatin resistance by transferring miR‐196a to target CDKN1B.^30^ CDKN1B was also identified as the second most common mutated gene in hairy cell leukemia, suggesting that CDNK1B may act as a novel cancer suppressor gene.^31^ Additionally, hemizygous deletions encompassing CDKN1B that suggest a role for p27 as a tumor suppressor in small intestine neuroendocrine tumors have been identified.^32^ In this study, we found that low expression of CDKN1B may be related to EOC progression and metastasis. More importantly, we found that patients with low CDKN1B expression exhibited a worse prognosis. However, for in vitro experiments, transfection of CDKN1B plasmid into miR‐221‐3p‐OE EOC cells could increase CDKN1B to a higher expression level than that induced by miR‐NC. These results confirmed that CDKN1B possessed a more complex upstream regulation mechanism, and miR‐221‐3p was an important but not the only factor in this mechanism.

In conclusion, our data revealed that TAMs‐derived exosomal miR‐221‐3p was one of the regulators in EOC progression through its ability to target CDKN1B. These findings suggest that these exosomal miRNAs may provide novel diagnostic biomarkers and therapeutic targets for EOC.

## CONFLICTS OF INTEREST

The authors declare no conflicts of interest for this article.

## AUTHOR CONTRIBUTIONS

XDL acquired the data and analyzed the interpretation of data. MLT developed the structure of the article and guided the selection of references. XDL and MLT wrote and reviewed the manuscript. Both the authors read and approved the final manuscript. The data used to support the findings of this study are included within the article and the supplement data, and they are also available from the corresponding author upon request.

## Supporting information

FigS1‐S2

Supplementary material
