# Supplementary material for: Exosomes released from M2 macrophages transfer miR‐221‐3p contributed to EOC progression through targeting CDKN1B
Source: Cancer Med. 2020 Jun 26;9(16):5976–88. doi: 10.1002/cam4.3252 (PMC7433826; doi:10.1002/cam4.3252)
Supplement: Supplementary file 1 — FigS1‐S2 [file CAM4-9-5976-s001.docx]

**Supplement data**

**
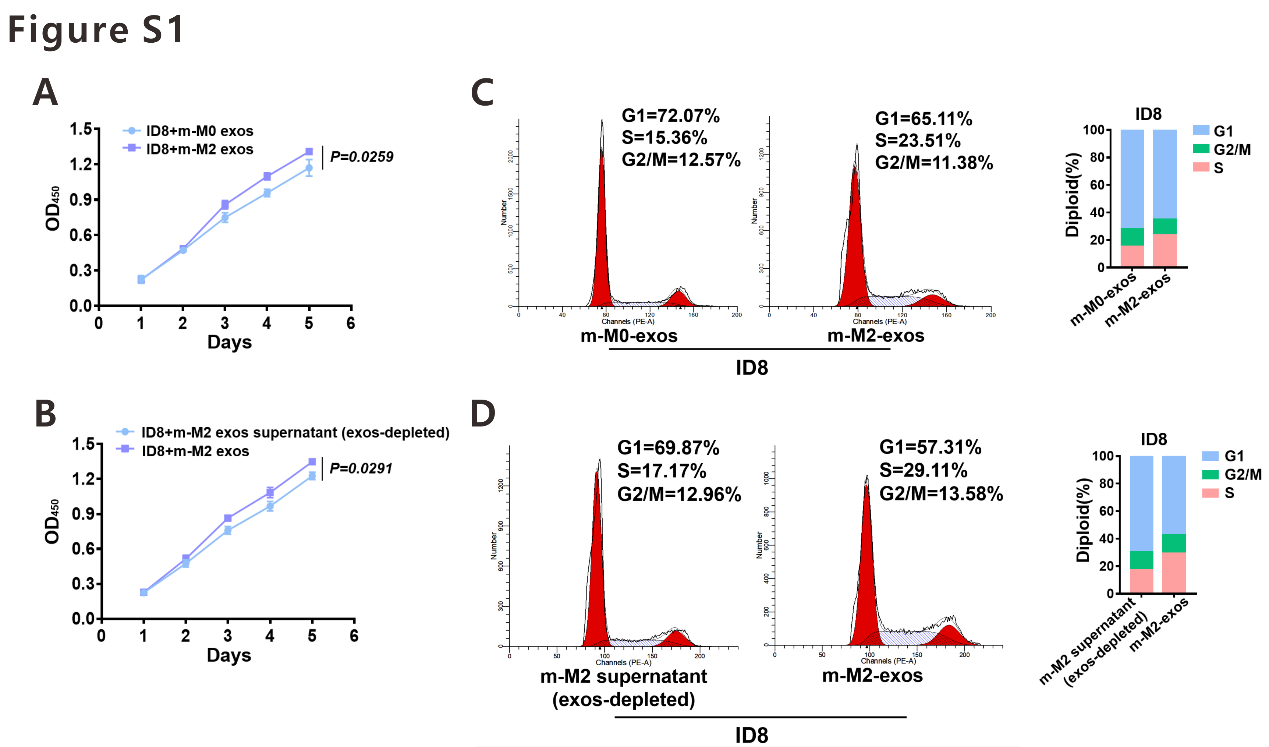
Figure S1.** **Exosomes from M2 macrophages played a crucial role in supernatants and contributed to ID8 cells proliferation and G1/S transition**

**(A)** CCK8 showed the proliferation of ID8 cells that co-cultured with M2/M0 exosomes. **(B)** CCK8 showed the proliferation of ID8 cells that co-cultured with M2 exosomes or exosomes-depleted supernatants. **(C)** Flow cytometry confirmed the G1/S transition of ID8 cells that co-cultured with the exosomes derived from M0/M2 macrophages. **(D)** Flow cytometry confirmed the G1/S transition of ID8 cells that co-cultured with M2 exosomes or exosomes-depleted supernatants.

**
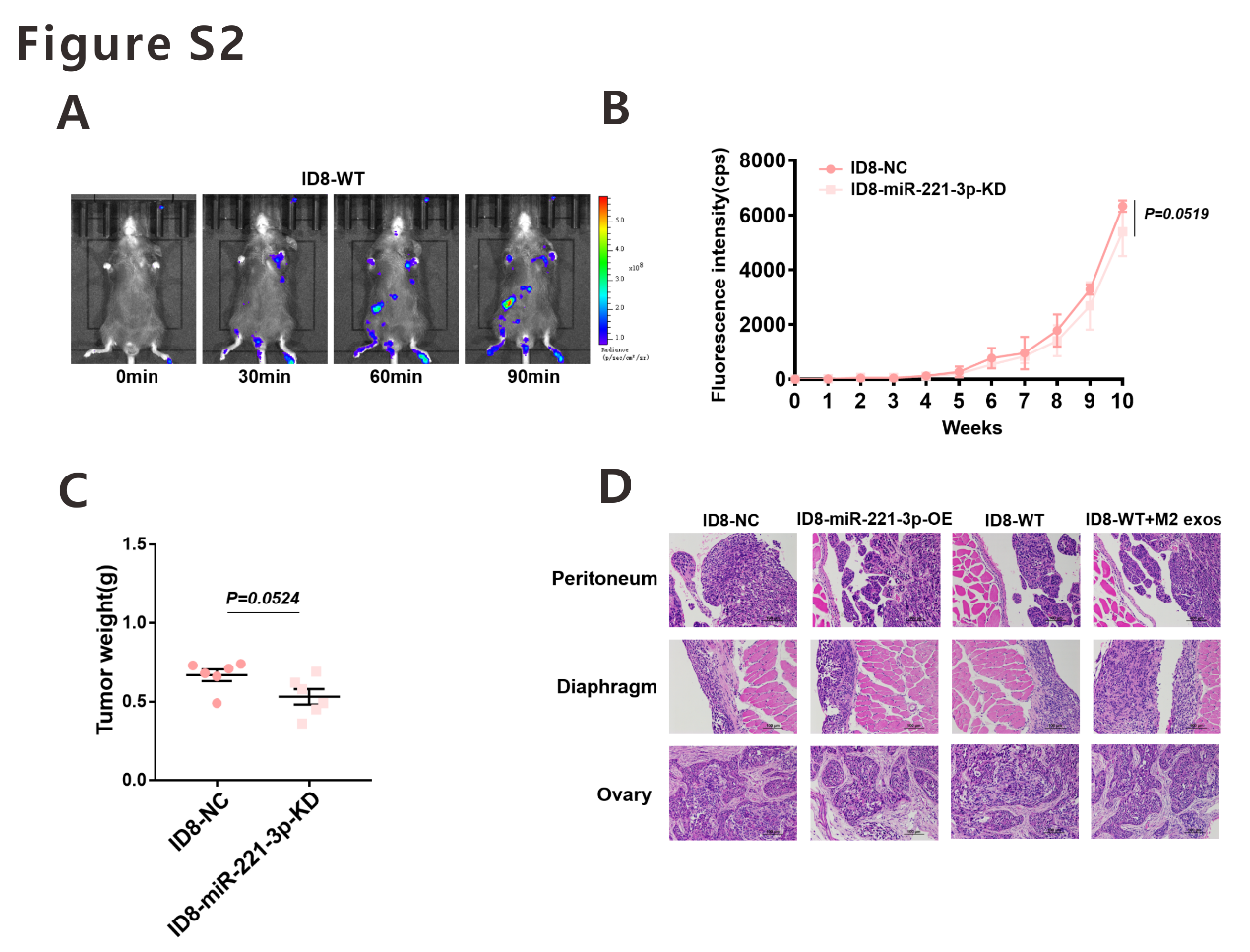
**

**Figure S2.** **M2 exosomes and miR-221-3p contributed to EOC progression *in vivo***

**(A)** The *in vivo* biodistribution for the intraperitoneally injected exosomes in ID8-WT group. **(B)** The trend in tumor fluorescence intensity (cps) of ID8-NC group and ID8-miR-221-3p-KD group at 1-10 weeks. **(C)** The tumor weight of ID8-NC group, ID8-miR-221-3p-KD group after mice were sacrificed. **(D)** Hematoxylin-eosin (H&E) staining of the peritoneum, diaphragm, ovarian from sacrificed mice in ID8-NC group, ID8-miR-221-3p-OE group, ID8-WT+M2 exos group. Scale bar, 100 μm.
